# Association of mSin1 with mTORC2 Ras and Akt reveals a crucial domain on mSin1 involved in Akt phosphorylation

**DOI:** 10.18632/oncotarget.18818

**Published:** 2017-06-28

**Authors:** Chien-An Yao, Sara Ortiz-Vega, Yun-Ya Sun, Chiang-Ting Chien, Jen-Hua Chuang, Yenshou Lin

**Affiliations:** ^1^ Department of Life Science, National Taiwan Normal University, Taipei, Taiwan; ^2^ Department of Family Medicine, National Taiwan University Hospital, Taipei, Taiwan; ^3^ Diabetes Unit and Medical Services and The Department of Molecular Biology, Massachusetts General Hospital and The Department of Medicine, Harvard Medical School, Boston, Massachusetts, USA

**Keywords:** mSin1, mTORC2, Akt, Ras, association

## Abstract

mSin1 is a unique component within the mammalian target of rapamycin (mTOR) complex 2 (mTORC2), which is responsible for cellular morphology and glucose metabolism. The association between mSin1 and other mTORC2 components, as well as their functions, has been explored previously; nevertheless, the mapping of the various binding domains of the components is lacking. Based on an evolutionary analysis of the gene, we constructed various fragments and truncated-forms of mSin1. We characterized the individual binding sites of mSin1 with its various partners, including mTOR, Rictor, Ras, and Akt. mTOR and Rictor bind to the amino acid (aa) 100-240 region of mSin1, which is different to the Ras binding site, the aa 260-460 region. A reciprocal examination found that mSin1 associated with the aa 2148-2300 region of mTOR, which is within the kinase domain, and with the carboxyl terminus of Rictor. Interestingly, Akt was found to associate with mSin1 in a region that slightly overlapped with the mTOR/Rictor complex binding site, namely aa 220-260. When only the Akt binding site was deleted from mSin1, phosphorylation of Akt S473 was greatly reduced. Furthermore, the association between Akt and mTOR can be regulated by serum, insulin and LY294002, but not by rapamycin or MAPK kinase inhibitors. Taken together, mSin1 would seem to act as a hub that allows mTORC2 to phosphorylate Akt S473. Our findings should facilitate future proteomic and crystallographic studies, help the development of dominant inhibitors and promote the identification of new drug targets.

## INTRODUCTION

The kinase mammalian target of rapamycin (mTOR) and its related signaling are closely associated with an organism's physiological and pathophysiological state, including cellular metabolism, survival, cell growth, proliferation, cell cycle, autophagy, aging, etc (see reviews [1–3]. Many drugs such as Sirolimus, Torin1 and PP242 have also been developed to target mTOR, as well as its downstream pathway, with the aim of targeting various cancers, diabetes, neurodegenerative diseases and aging [4–8]. The multiplicity of functions associated with mTOR are the partial result of the two physically distinct and separate hetero-oligomeric complexes found in cells; these are designated mTOR complex 1 (mTORC1) and mTOR complex 2 (mTORC2). mTORC1 contains three core polypeptides mTOR, Raptor (regulatory protein associated with mTOR) and mLST8 (mammalian lethal with Sec13 protein 8). On the other hand, the four core proteins that make up the mTORC2 complex are mTOR, mLST8, Rictor (rapamycin insensitive companion of mTOR) and mSin1 (mammalian SAPK interacting protein 1) [3]. In contrast to mTORC1, the upstream pathways, regulation and roles of mTORC2 are much less understood. Thus far, Akt/PKB, SGK1, and PKCζ are the three substrates known to be directly phosphorylated by mTORC2 as part of its downstream signaling [9–12].

Within the mTORC2 complex, mSin1, mammalian orthologue of Sin1, is indispensible to Akt phosphorylation [13–15]. The loss of Sin1 in *S. pombe* results in both impaired phosphorylation of the transcription factor Atf1 and a stress-sensitive phenotype that can be rescued by a fusion protein encoding the C-terminal 182 amino acids of chicken Sin1 [16]. Subsequent studies in mammalian cells have identified mSin1, also called Mip1, to be a MEKK2 binding protein that also binds SAPK/JNK [17, 18]. Interestingly, Schroder et al reported that mSin1 contains Raf-like Ras-binding domains (RBD) that are responsible for the binding to Ras [19]. Recently, it has been inferred that the N-terminus of mSin1 is responsible for the binding of mSin1 to mTORC2 [20]. While it has been clearly shown that mSin1 is an intrinsic component of mTORC2, published studies on mSin1 have not addressed in detail the regions involved in the binding of mSin1 to its various partners.

Mapping the binding domains between proteins has important implications; these include determining details of the binding mechanism, identifying possible specific activators/inhibitors, and facilitating the development of relevant drug targets. Based on a bioinformatics analysis of the mSin1 evolution [21], we constructed a number of different fragments of mSin1 covering different Sin1 conserved domains (SCD) in order to study the various associations within the mTORC2 complex. Our findings not only have created a plausible three-dimension relationship among these proteins, but should also greatly help the development of new therapeutic strategies for the treatment of mTOR related diseases, in particular various cancers.

## RESULTS

### mSin1 binds to the kinase domain aa 2148-2300 of mTOR

**Figure 1:**
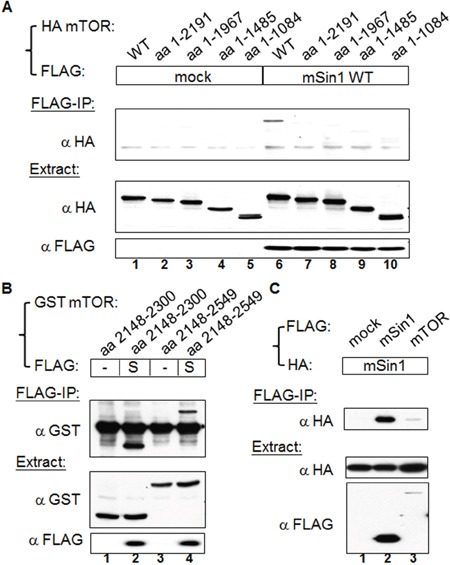
mSin1 binds to the kinase domain of mTOR **(A)** HEK 293T cells were co-transfected with indicated FLAG/FLAG-mSin1 and HA-mTOR plasmids (full length, aa 1-2191, 1-1967, 1-1485, and 1-1084). The expressed proteins from the lysate were subjected to FLAG antibody IP. **(B)** HEK 293T cells were co-expressed with FLAG-mSin1 wild-type (S) and GST-mTOR fusion proteins (aa 2148-2300 and aa 2148-2549). The cells were lysed and the supernatants were performed FLAG antibody IP. **(C)** HEK 293T cells were co-transfected with indicated FLAG/FLAG-mSin1/FLAG-mTOR and HA tagged mSin1. The expressed proteins from the lysate were subjected to FLAG antibody IP and Western blot analysis. Anti-FLAG, anti-HA, or anti-GST antibodies were used to detect appropriate proteins in the total lysates, the IP samples, and pull-down samples. The blots are representative of one experiment repeated twice.

Since mTOR is the major enzymatic molecule in the mTORC2, we initially examined the mSin1 binding site within mTOR that retained its full length of 2549 amino acids. All amino terminus mTOR fragments shorter than aa 2191 did not bind, whereas the wild-type of mTOR did bind (Figure 1A); interestingly and logically, it was found that aa 2148-2549 of mTOR did associate with mSin1 (Figure 1B, lane 4). We further found that it is the kinase domain, aa 2148-2300, of mTOR that binds to mSin1 (Figure 1B, lane 2). Moreover, as shown in Figure 1C, FLAG tagged mSin1 is able to pull down HA tagged mSin1. Binding between FLAG-mTOR and HA-mSin1 was also included as a control. Since mTOR is capable of forming multimers, most likely dimers [22], we believe that our findings indicate that the association might be via either direct interaction or perhaps via indirect interaction that is mediated by mTOR dimerization.

### mSin1 binds to the carboxyl terminus aa 1181-1708 of Rictor

**Figure 2:**
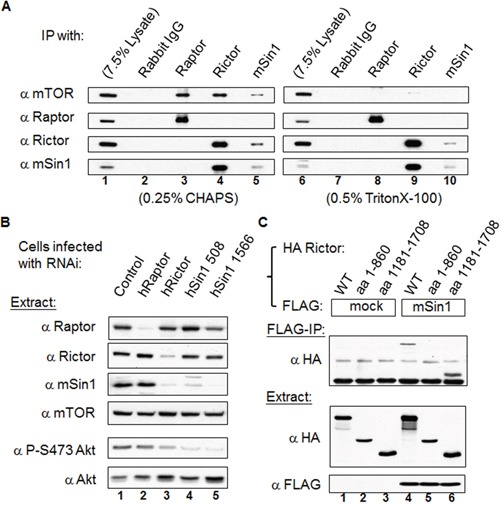
mSin1 forms a stable complex with Rictor independent of mTOR and binds to the carboxyl terminus of Rictor **(A)** HEK 293T cells were lysed in buffer containing 0.25% CHAPS (left panels) or 0.5% Triton X-100 (right panels). Antibodies against mTORC1 or mTORC2 were used to perform IP or reciprocal IP. **(B)** Viral particles containing Raptor, Rictor or two types of mSin1 RNAi were used to infect HEK 293T cells. Cells infected with luciferase RNAi were included as a control. The lysates were processed by Western blot analysis to detect the various proteins, Akt abundance and the phosphorylation of Akt S473 (P-S473 Akt). **(C)** HEK 293T cells were co-transfected with the indicated FLAG/FLAG-mSin1 and HA tagged Rictor plasmids (full length, aa 1-860 and 1181-1708). The expressed proteins were subjected to FLAG antibody IP. Anti-FLAG and anti-HA antibodies were used to detect the appropriate proteins in the total lysates and in the IP samples. The blots are representative of one experiment repeated twice.

We confirmed the endogenous association and the effects of detergents on the mSin1 and various mTOR complex component relationships [23]. As shown in the left panels of Figure 2A, Raptor, Rictor, and mSin1 antibodies individually are able to immunoprecipitate (IP) mTOR, whereas mSin1 can only co-precipitate with Rictor and not with Raptor (lane 5). Conversely, Rictor antibody is able to pull-down mSin1 (lane 4), whereas Raptor antibody binds to neither of them. In parallel, the same experiment was executed in the presence of TritonX-100, as seen in the right panels of Figure 2A. Interestingly, while mTOR was washed away when the Rictor and mSin1 IPs were carried out, Rictor and mSin1 was still able to pull down each other (lane 9 and 10). This indicates that Rictor and mSin1 are able to form a complex independent of mTOR. Furthermore, it has been previously showed that the RNAi of Rictor and of mSin1 is able to bring about a decrease in PKB/Akt S473 phosphorylation independently [12, 14]. In addition, confirming these findings, it was found that both Rictor and mSin1 proteins are decreased in Rictor knock-down cells (Figure 2B). Nevertheless, only the abundance of mSin1 protein, but not the abundance of Rictor protein, is affected in mSin1 RNAi knockdown cells. This implies that the interaction between mSin1 and Rictor might not be a mutual dependent one. We further examined the mSin1 binding site within Rictor that was full length at 1708 amino acids. Based on SMART analysis (http://smart.embl-heidelberg.de/), Rictor protein contains few identifiable regions except for a RasGEF N domain in the region aa 743-857. Thus we simply divided this protein into three fragments based on a bioinformatics analysis. Unfortunately the aa 861-1180 fragment was found not to be expressed at all. However, using the two other fragments, as shown in the Figure 2C, we found that only the aa 1181-1708 fragment was able to bind to mSin1. Hence, it would seem that the carboxyl terminus of Rictor, but not amino terminus, is involved in binding to mSin1.

### mSin1 binds to mTOR/Rictor via mSin1 aa 100-240

**Figure 3:**
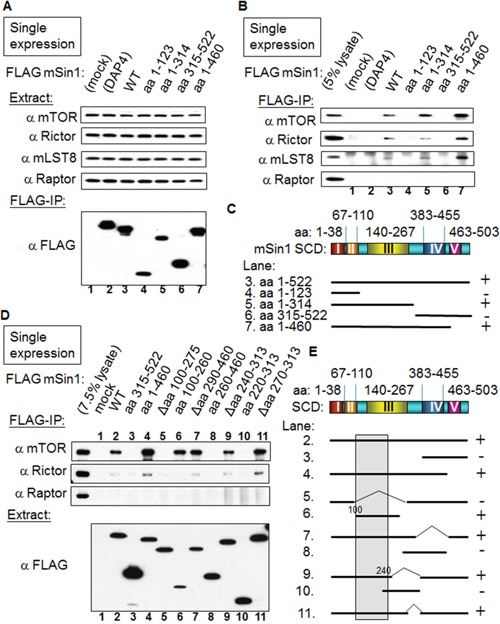
mSin1 aa 100-260 binds to mTOR/Rictor **(A)** and **(B)** HEK 293T cells were individually transfected with various forms of FLAG-mSin1 (aa 1-123, 1-314, 315-522, 1-460, and wild-type). The expressed proteins from the lysate were subjected to FLAG antibody IP. Anti-mTOR, Rictor, mLST8, and Raptor antibodies were used to assess/detect the appropriate protein levels in the total lysates and in the IP samples. Both FLAG only (mock) and FLAG tagged death associated protein 4 (FLAG-DAP4) were included as negative controls. **(C)** The binding between the various fragments of mSin1 and the different endogenous proteins is summarized. The recovery of the mTOR/Rictor/mLST8 together with mSin1 fragments including different Sin1 conserved domains (SCD) is indicated on the right as either + or -. **(D)** After more experiments of trials and errors, HEK 293T cells were individually transfected with the indicated FLAG-mSin1 WT or FLAG-mSin1 fragments. The expressed proteins from the lysate were subjected to FLAG antibody IP and Western blot analysis. Indicated antibodies were used to detect the appropriate associated proteins in the total lysate and in the IP. **(E)** The recovery of endogenous mTOR with the fragments of mSin1 is summarized and indicated on the right as either + or -. The blots are representative of one experiment repeated twice.

We began by mapping the binding sites of mSin1 that was full length at 522 amino acids in relation to the various individual components found within the mTORC2 complex. Initially, HEK 293T cells were co-transfected with tagged mSin1 along with individual tagged recombinant genes (Supplementary Figure 1). We soon found that a single transfection of FLAG tagged mSin1 followed by FLAG IP showed the same level of robust binding of the various endogenous partners as the ones obtained using co-transfection. Therefore, the associated mTORC2 components shown in Figure 3 are the endogenous proteins detected by blotting using appropriate individual antibodies. The expression of the individual endogenous proteins as well as FLAG-mSin1 full length and its various fragments are shown in the Figure 3A. As shown in the Figure 3B, mSin1 aa 1-314 and aa 1-460, but not aa 1-123, are each capable of associating with mTOR/Rictor/mLST8. The association between mSin1 and the endogenous mTORC2 components is schematically presented in Figure 3C. The findings suggest that the binding site is within aa 123-314. After testing over several rounds and adjusting/constructing proteins consisting of a range of different regions within mSin1, it was possible to map the shortest fragment to the range aa 100-260 within mSin1 as seen in Figure 3D. All the mutants that encompass this region were found to be capable of binding to endogenous mTOR (Figure 3D). Conversely, mutants that lack this region, for example Δaa 100-275 in lane 5, were unable to bind endogenous mTOR. Based on these findings, the fragment of mSin1 involved in endogenous Rictor binding would also seem to be located within the region aa 100-260 because the pattern obtained is similar to that for mTOR binding. A Raptor blot is included as a comparison. However, since the Rictor pull-down is not so obviously visualized using the mSin1 fragment aa 100-260, in contrast to other variants, it is possible that Rictor might need a larger domain, such as aa 1-260 of mSin1, to form a tight association. The associations between the various mSin1 fragments and mTOR/Rictor are summarized in Figure 3E. We infer from these results that the shortest region of mSin1 capable of undergoing endogenous mTOR binding is the area around aa 100-240 (lanes 6, 9 in Figure 3E).

### mSin1 binds to active HaRas, but not other active Ras superfamily members, via the mSin1 aa 260-460 region

**Figure 4:**
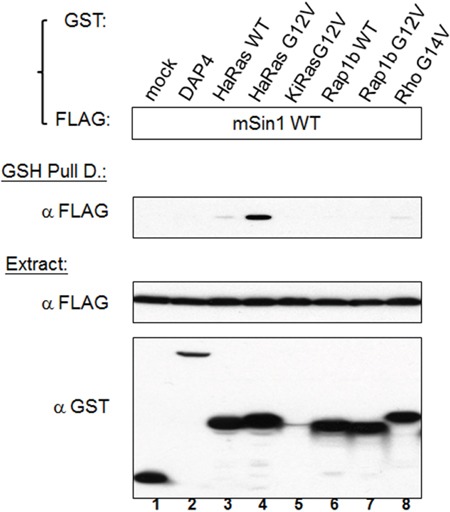
Active Ras, but not other active forms of members of the Ras superfamily, is able to bind to mSin1 HEK 293T cells were co-transfected with the indicated FLAG-mSin1 WT and with various GST tagged plasmids, namely HaRas WT, HaRas G12V, KiRas G12V, Rap1b WT, Rap1b G12V and Rho G14V. The expressed proteins from the lysate were analyzed by Western blotting using anti-FLAG and GST antibodies. After the lysates were subjected to GSH pull down, anti-FLAG antibody was used for the Western blot analysis. The blots are representative of one experiment repeated twice.

**Figure 5:**
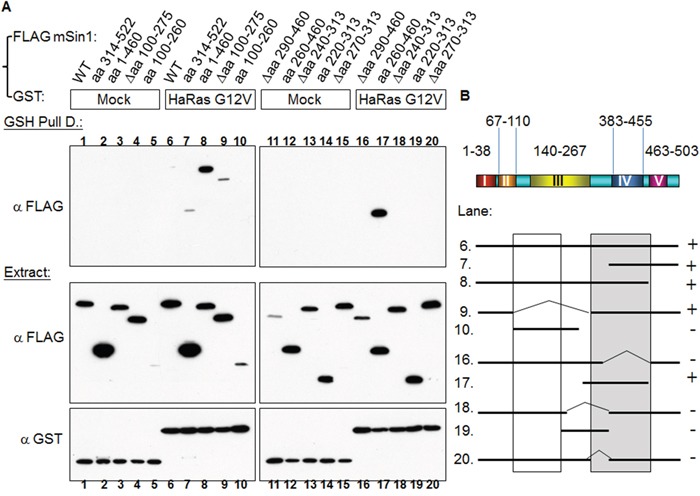
HaRas G12V binds to mSin1 aa 260-460, a region that is different to the one involved in mTORC2 binding **(A)** HEK 293T cells were co-transfected with GST/GST-HaRas G12V and the various indicated FLAG-mSin1 fragments. A portion of each extract was used to examine the expression level of the GST and FLAG tagged proteins. The majority of each lysate was subjected to GSH pull down. Anti-FLAG antibody was used to detect the associated proteins after the pull-down. **(B)** The binding between fragments of mSin1 and the recombinant HaRas G12V proteins is summarized here. The recovery of the HaRas G12V association is indicated on the right as either + or -. The blots are representative of one experiment repeated twice.

Sin1 has been reported to preferentially bind to the GTP-bound form of Ras in *Dictyostelium* [24]. Schroder et al. also found that a similar effect occurred in mammalian cells [19]. Intriguingly, we found that HaRas G12V shows a strong association with mSin1, but that there was no similar association with either Rap1b G12V or Rho G14V, as is shown in Figure 4. We then mapped the binding site and found that the mSin1 aa 260-460 is able to associate with HaRas G12V (Figure 5A). No binding occurred when the mSin1 mutant lacking this region was used. However, the expression of the mSin aa100-260 is quite low compared to the other mSin mutants. As a result of this, we place a caveat on the above and suggest that instability may be the reason for the low expression. The association between the various mSin1 fragments and active HaRas is summarized in Figure 5B. Based on these findings, we believe that the binding site for mSin1, when it associates with active HaRas, is located within the aa 260-460 region which contains SCD IV (i.e. aa 383-455). Furthermore, we also have shown that a mutant lacking aa 290-460 significantly impairs the phosphorylation of SAPK (Supplementary Figure 2).

### mSin1 binds to Akt/PKB via mSin1 aa 220-260

**Figure 6:**
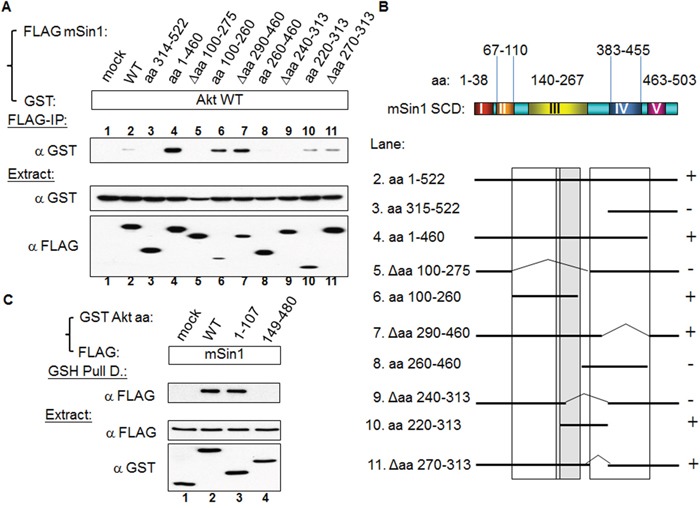
Akt binds to mSin1 aa 220-260, a region between the mSin1 binding site for mTOR/Rictor and for HaRas **(A)** HEK 293T cells were co-transfected with the various indicated FLAG-mSin1 fragments and GST-Akt WT. A portion of each extract was used to examine the expression levels of the FLAG and GST tagged proteins. The majority of each lysate was subjected to FLAG antibody IP. Anti-GST antibody was used to detect the present of the various associated proteins after the IP. **(B)** The binding between the various recombinant fragments of mSin1 and recombinant Akt protein is summarized. The recovery of Akt association is indicated on the right either as + or -. **(C)** HEK 293T cells were co-transfected with the indicated FLAG-mSin1 and GST tagged Akt plasmids (full length, aa 1-107 and 149-480). The expressed proteins were subjected to GST pull-down. Anti-FLAG and anti-GST antibodies were used to detect the appropriate proteins in the total lysates and in the pull-down samples. The blots are representative of one experiment repeated twice.

mTORC2 has been proposed to function as a PDK2 and phosphorylate Akt at S473 [12, 14]. Based on this we examined whether Akt is able to associate with mSin1. Using the constructs made up of the various regions of mSin1, we mapped the Akt binding site to be located in the region aa 220-260 of mSin1 (Figure 6A). Any fragments, such as aa 220-313, that include this region, also show an association (lane 10). By way of contrast, proteins lacking this region, for example Δaa 240-313 in lane 9, are unable to bind Akt. The association between the various mSin1 fragments and Akt is summarized in Figure 6B. These findings imply that the binding site within mSin1 for Akt is located in the region aa 220-260 (lanes 6, 9 and 10 in Figure 6B). This region is quite distinct from the main binding sites for the other components that form the mTORC2 complex and involved in active HaRas association. In addition, we also reciprocally mapped where the sites of Akt binds to mSin1. As shown in Figure 6C, Akt aa 1-107, but not aa 149-480, binds to mSin1. This indicates that it is the pleckstrin homology domain (PH domain) of Akt, and not the kinase domain, which is capable of binding to mSin1.

### The association between Akt and mTOR is regulated by serum, insulin and the PI3K inhibitor LY294002

**Figure 7:**
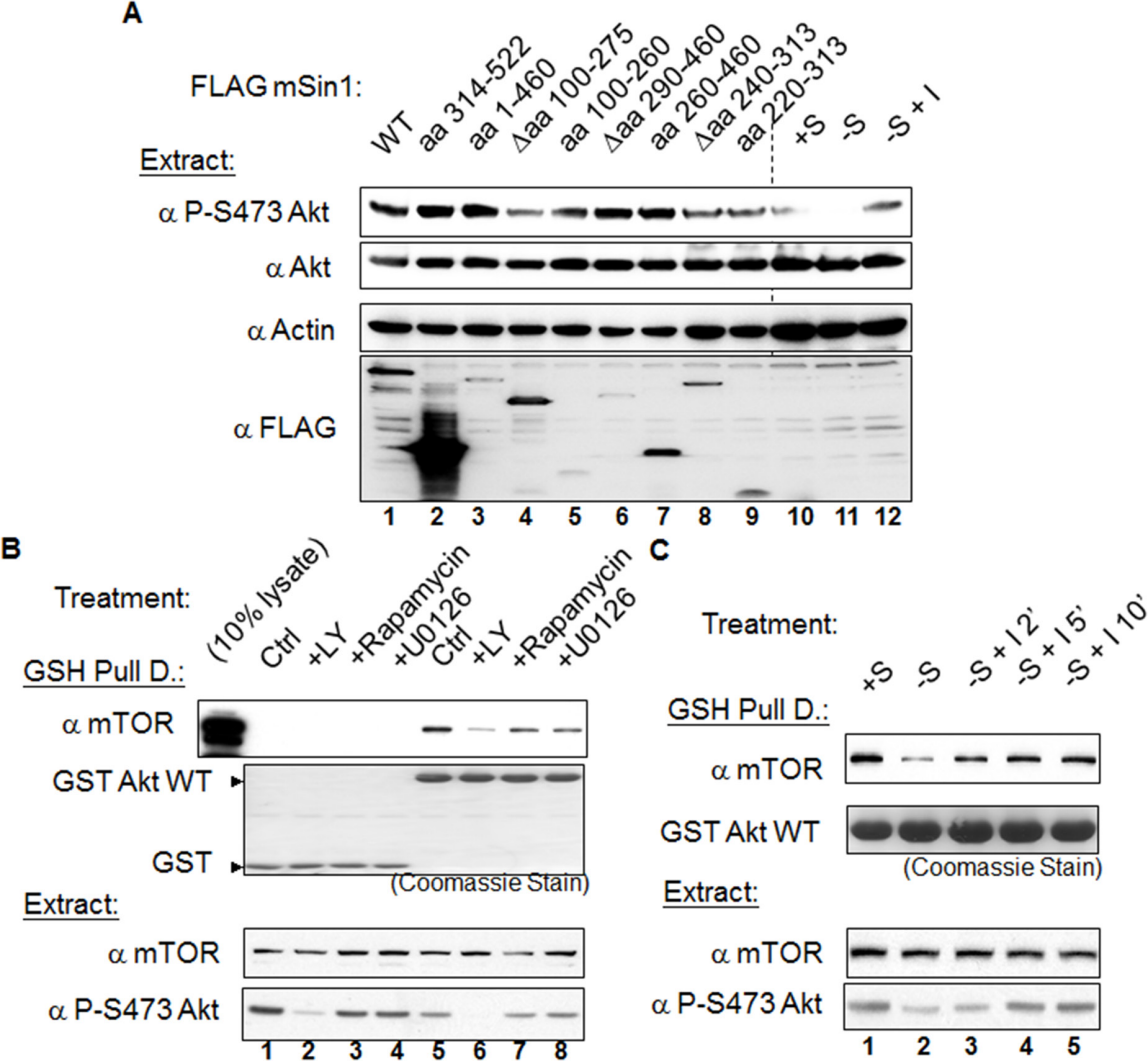
The mSin1 aa 100-275, namely the binding sites for mTORC2 and Akt, is necessary for phosphorylation of Akt at the S473 site The association between mTOR and Akt can be regulated by serum, insulin and the presence of a PI3K inhibitor. **(A)** HEK 293T cells were only transfected with the various indicated FLAG-mSin1 fragments. Cells were lysed in the lysis buffer containing 0.5% TritonX-100. Anti-phosphorylated Akt S473 antibody was used to detect the functioning of these mSin1 fragments. The last three lanes (lane 10, 11, and 12) were cells treated with normal serum (+S), serum withdrawal (−S), or insulin stimulation after serum withdrawal (−S+I) and the Akt signaling changes were used as the antibodies efficacy controls. **(B)** HEK 293T cells were transfected with either GST or GST Akt. Twenty-four hours after transfection, the cells from the master culture were split into individual plates so that the starting material was the same. Sixteen hours after the split and before the cells were harvested, the cells were treated with vehicle DMSO, 20 μM LY294002, 1 μM rapamycin, or 10 μM U0126 for 1 h. **(C)** HEK 293T cells were transfected with GST Akt. Twenty-four hours after transfection, the cells from master culture were split into individual plates so that the starting material was the same. The cells were then either continuously cultivated in medium containing serum (+S) or in serum depletion medium (−S), both for an additional 16 h. Immediately before harvesting these groups of cells, insulin 1 μM was added into the medium for an additional 2, 5, or 10 min. After processing by GST pulling down and Western blot analysis, antibodies such as anti-GST, mTOR, and phosphorylated S473 Akt were used to detect the signal. The blots are representative of one experiment repeated twice

We next examined the possible binding-associated functional relationship between mSin1 and mTORC2. Overexpression of mSin1 Δaa 100-275 (lane 4) resulted in a dramatic decrease in phosphorylation at endogenous Akt S473 when the expression of these fragments was at similar level (Figure 7A). In the same experiment, HEK 293T cells were subjected to normal serum (lane 10) or serum starvation for 16 h followed by treatment with 100 nM-1 μM insulin for 0 (lane 11) and 20 min (lane 12). We used these treatments to verify the authenticity and specificity of the antibodies used. This finding implies that the mTORC2 complex and its substrate Akt might need to bind to a particular region on mSin1 in order to assemble a functional unit. On the other hand, fragments lacking mTOR/Akt binding, such as mSin1 aa 314-522, do not have such an effect. We interpret this to mean that mSin1 Δaa 100-275 is a special mutant that fulfills the criteria of having a dominant negative function, while the other mutants do not have this ability.

In addition to the above, we also examined whether the enzyme-substrate complex formed between mTORC2 and Akt undergoes regulation by various factors. Using a pull-down approach, we found that GST Akt is able to pull down endogenous mTOR (Figure 7B). The association between mTOR and Akt is disrupted by treatment with the PI3 kinase inhibitor LY294002, but is not affected by the mTOR inhibitor rapamycin or the MAPK inhibitor U0126 (Figure 7B). Furthermore, the amount of associated mTOR is decreased when the cells are subjected to the overnight serum withdrawal. Interestingly, this attenuation of the association can be reversed by adding insulin back into the culture medium (Figure 7C).

## DISCUSSION

During the present study, we have pinpointed the binding sites that allow interactions between mSin1 and mTOR, Rictor, Ras and Akt. We found that it is the kinase domain of mTOR, carboxyl terminus of Rictor, and PH domain of Akt that bind to mSin1. The binding site for Akt on mSin1 is adjacent to the site for mTOR/Rictor binding and is crucial for the phosphorylation of Akt at S473 by the mTOR kinase. Importantly, the association/dissociation between Akt and mTOR can be regulated by serum and insulin and this is mediated via the PI3K pathway.

Frias et al observed that the mSin1 isoform 4, which lack the first 192 amino acid residues, does not bind to mTORC2, while the other isoforms do bind [13]. They proposed that aa 1-192 within mSin1 allows binding to mTORC2. However, we found initially that the region aa 1-123, which contains SCD I and SCD II, is not able to associate with mTOR and/or mLST8 during our initial attempt to map the binding sites (Supplementary Figure 1). This discrepancy was part of our rationale for initiating this study. After more detailed mapping of the region, we found that the region aa 100-260 of mSin1 is the one able to bind to mTORC2 and this covers the SCD III region aa 140-267. The aa 100-260 of mSin1 also corresponds to the CRIM region aa 137-266, a region named after “conserved region in the middle” of all proteins in the Sin1 family [25]. This CRIM region is also known to associate with PKCα and PKCε [20]. Specifically, Cameron et al mapped the site to be within the aa 193-320 region of mSin1. In addition, we found the binding site between Akt and mSin1 is within the aa 220-260 region of mSin1. Since the direct substrates of mTORC2 are thought to be Akt [12], PKCs [11, 26], and SGK1 [9], our findings suggest that there might be only trivial differences among the bind sites for these AGC kinases when they interact with the SCD III region. Interestingly, the phenomenon that mSin1 aa 1-460 always has a better binding ability toward mTOR/Rictor/Akt compared to mSin1 wild-type indicates that the SCD V (aa 464-503) might have some inhibitory effect on this association.

When mSin1 aa 100-260 is absent, mSin1 is unable to bind to mTORC2 and Akt. Although mTOR is a large polypeptide and has multiple domains within the protein, mSin1 seems to associate with the mTOR kinase domain. Thus mSin1 might function as a scaffold protein that brings Akt, the substrate of mTOR, into a compact conformation that is able to be phosphorylated at its S473 residue. The binding sites between mTOR/Rictor and Akt on mSin1 are adjacent, which seems likely to create a conformation that allows mTORC2 to function as a PDK2. Supporting this hypothesis, mSin1 Δaa 100-275, which lacks the Akt binding site, does show a dramatic decrease in Akt S473 phosphorylation. This could provide fundamental and useful information for the solving of the three-dimension structure of the mTORC2. In addition, mSin1 Δaa 100-275 demonstrates a unique character with respect to decreasing Akt S473 phosphorylation with no other fragments have a similar effect. Therefore, the criteria for mSin1 mutants to be a dominant negative fragment in terms of Akt phosphorylation might be that they need to strictly fulfill all associations except for those with the mTOR and Akt proteins. Knowledge of this specificity probably enhances the possibility of developing inhibitory drugs that are able to block the phosphorylation and/or activation of the oncogene Akt.

Sin1 in *Dictyostelium* binds to RasG Q61L, but not RasG S17N [24]. This mutant in the Ras binding domain (RBD) has been found to greatly impair *Dictyostelium* chemotaxis/aggregation [27]. As part of the present study we have found that active HaRas binds to mSin1 at a totally separated domain to that of mTOR/Rictor and Akt. It has been reported that mSin1 contains a Ras binding domain and a pleckstrin homology domain and is able to suppress Ras signaling [19]. Therefore, the different binding sites of mTOR/Rictor/Akt/HaRas on mSin1 might plausibly create a range of different complexes in response to various different stimuli, which would in turn result in mSin1 being able to mediate control of multiple pathways. Recently, it has been reported that Sin1γ, a mSin1 isoform, is able to interact with the components that form the mTORC2 complex, but does not seem to participate in the functioning of mTORC2 with respect to the phosphorylation of Akt S473 [28]. Obviously, it is clear that the different regions as well as the various isoforms of mSin1 are likely to contribute to a range of effects on signal transduction within cells.

Typically, protein-protein interactions are a central concept of signaling transduction, although protein-lipid and other forms of interaction are also likely to play roles [29]. It has been proposed that Akt activation by growth factors is due to the recruitment of PDK1 and Akt to the membrane via their PH domains [12, 30, 31]. At the membrane, Akt T308 and S473 were respectively phosphorylated by PDK1 and mTORC2. Here, we have provided a further possibly regulatory mechanism whereby a decrease in Akt S473 phosphorylation caused by serum withdrawal might simply occur via the dissociation of Akt from the mTORC2.

**Figure 8:**
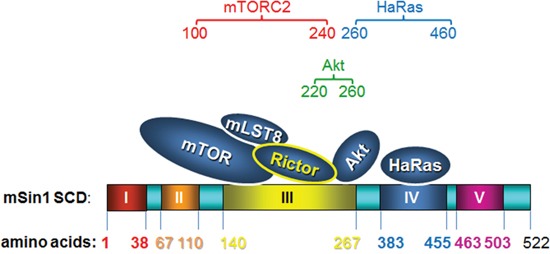
A cartoon schematically presenting how mSin1 associates with its various partners It is the kinase domain of mTOR, the carboxyl terminus of Rictor, and the pleckstrin homology domain of Akt that bind to mSin1. Based on these findings in terms of association between the various proteins and the various fragments of mSin1, we suggest that mTORC2, Akt, and HaRas, respectively, associate with mSin1 aa 100-240, mSin1 aa 220-260, and mSin1 aa 260-460.

In summary, we provide in this study a structure/function analysis describing the proteins interaction between mTORC2, Ras, Akt and mSin1. The association between mTOR and Akt can be regulated by insulin and serum. Such enzyme-substrate binding is blocked by a PI3K inhibitor, but not by either rapamycin or a MAPK inhibitor. The binding process is depicted schematically as Figure 8, which shows how mSin1 bind to its various partners using different regions within the protein. These findings provide fundamental information that will help our understanding of the three-dimension topography of the mTORC2, especially how the enzyme, the scaffolding and the substrate(s) interact. The results also facilitate our greater understanding of this complex proteomic/signaling network. Furthermore, the regions we have mapped should help with the development of drug targets and/or inhibitors that are relevant to a number of diseases including cancer.

## MATERIALS AND METHODS

### Reagents

The materials/reagents were purchased from the companies indicated in parentheses: Dulbecco's modified Eagle's medium (DMEM, Gibco-BRL-Life Technologies, NY, USA); fetal bovine serum (FBS, Thermo Scientific, Utah, USA); protein G-agarose and protein A-agarose, antibody against GST (Santa Cruz Biotechnology, CA, USA); antibodies against Akt serine 473 (S473) and pan Akt (Epitomics, CA, USA); antibody against HA (Covance, NJ, USA); antibody against FLAG (Sigma, MO, USA); horseradish peroxidase anti-rabbit (Jackson, PA, USA); and anti-mouse secondary antibody (Jackson, PA, USA). The antibodies against mTOR, Raptor, and mLST8 have been described previously [32]. The antibodies against Rictor and mSin1 were respectively generated by introducing DNA fragments into the pGEX GST plasmid corresponding to aa 1508-1708 of Rictor and aa 314-522 of mSin1 and expressing them in the BL21(DE3) pLysS strain of *E. coli*. The proteins were purified using GSH agarose and the two eluates were then used as antigens to immunize rabbits. The injection and maintenance of rabbits were executed by Cocalico Biologicals, Inc (Reamstown, PA, USA). The same fragments of Rictor and mSin1 were also cloned individually into the PinPointXa vector (Promega, WI, USA) to generate biotinylated proteins in bacteria. The antibodies in rabbit sera were affinity purified using the biotin-avidin resin system by following the manufacturer's instruction (Promega, WI, USA). All other chemicals were purchased from Sigma (MO, USA) unless otherwise indicated.

### Plasmid construction

The mouse Sin1 gene was subcloned from a cDNA clone (MGC:106246, IMAGE: 6392317) into a series of different mammalian expression vectors after PCR in which the 5′ primer used GAATTCTATGGCCTTCTTGGACAATCCAACT and the 3′ primer used GCGGCCGCTCACTGCTGCCCTGATTTCTTCTC. The wild-type and various mutants of mTOR, Rictor, mSin1, Ras and Akt genes were also subcloned into a series of different mammalian expression vectors. Each of these vectors produces proteins containing different types of tag, namely FLAG, HA, and GST. All the constructs were confirmed by DNA sequencing.

### Cell culture and transfection

HEK 293T cells were cultivated in DMEM supplemented with 10% FBS and penicillin/streptomycin in a 5% CO_2_ at 37°C in an incubator. When transfection was performed, the cells were plated at a density of 3.5 × 10^6^ per 10 cm dish and transfected 5 h later using a total of 10 μg of DNA and 30 μl of Lipofectamine per dish by following the manufacturer's instructions (Invitrogen, CA, USA). Drop-wise addition of the DNA mixture onto each 10 cm dish was executed and then the dishes were incubated for additional 36 to 48 h.

### Cell lysis, immunoprecipitation, and Western blotting

Frozen HEK 293T cells was lysed in the lysis buffer (20 mM Tris base, pH 7.9, 20 mM NaCl, 1 mM EDTA, 5 mM EGTA, 20 mM β-glycerophosphate, 1 mM dithiothreitol, 1 mM phenylmethylsulfonyl fluoride, 25 nM calyculin A, 0.25% (w/w) CHAPS, 1 tablet/50 ml of protease inhibitor (Roche Molecular Biochemicals)). Next, 2 μg of IgG, Rictor, Raptor or FLAG antibodies were mixed with protein A/G-agarose at 4°C for 1 h and then washed three times in lysis buffer. The cell lysates were centrifuged at 13,500 rpm for 10 min and aliquots of the supernatants, containing equal amounts of protein as measured by Bradford assay (Bio-Rad), were added to 15 μl of settled antibodies-agarose beads and incubated at 4°C for 2 h. The beads were washed three times with 1 ml of lysis buffer, twice with 1 ml of lysis buffer containing 0.5 M NaCl, and then once with 1 ml of lysis buffer. Finally the adsorbed proteins were released using SDS sample buffer and incubation at 95°C for 10 min. The released proteins were separated by SDS-polyacrylamide gel electrophoresis (SDS-PAGE) followed by transferring onto a polyvinylidene difluoride (PVDF) membrane. The various membranes were probed with the appropriate specific antibodies as indicated. The blots were visualized using horseradish peroxide-conjugated secondary antibody followed by chemiluminescence using the manufacturer's protocol (Millipore, MA, USA).

### RNAi viral particle preparation

For RNAi viral particle preparation, bacterial clones or plasmids containing short hairpin RNA oligonucleotides of the target genes were obtained from the National RNAi Core Facility (Genomic Research Center, Academia Sinica, Taipei, Taiwan) and have been partially described previously [33]. The functional clones and corresponding effective target sequences of the control, of Raptor, of Rictor, and of mSin1 were verified and are: luciferase (control), 5′CGCTGAGTACTTCGAAATGTC, Raptor sequence, 5′GGCTAGTCTGTTTCGAAATTT, Rictor sequence, 5′ACTTGTGAAGAATCGTATCTT, the mSin1 508s1c1 sequence 5′CAGTCGATATTACCTCAAGTT, and another mSin1 1566s1c1 sequence 5′CTAAGCAATCACGACTATAAA. The RNAi plasmids were co-transfected with two other viral packaging plasmids, pCMV8.91 and pMD.G, into the HEK 293T cells. The virus-containing medium was collected after transfection for 40 h and concentrated by ultracentrifugation at 25,000 rpm for 4 h. The pellets were then dissolved in PBS buffer and stored at −80°C until use.

## SUPPLEMENTARY FIGURES


