# Supplementary material for: Association of mSin1 with mTORC2 Ras and Akt reveals a crucial domain on mSin1 involved in Akt phosphorylation
Source: Oncotarget. 2017 Jun 28;8(38):63392–404. doi: 10.18632/oncotarget.18818 (PMC5609931; doi:10.18632/oncotarget.18818)
Supplement: Supplementary file 1 [file oncotarget-08-63392-s001.pdf]

Association of mSin1 with mTORC2 Ras and Akt reveals a crucial domain on mSin1 involved in Akt phosphorylation

SUPPLEMENTARY MATERIALS

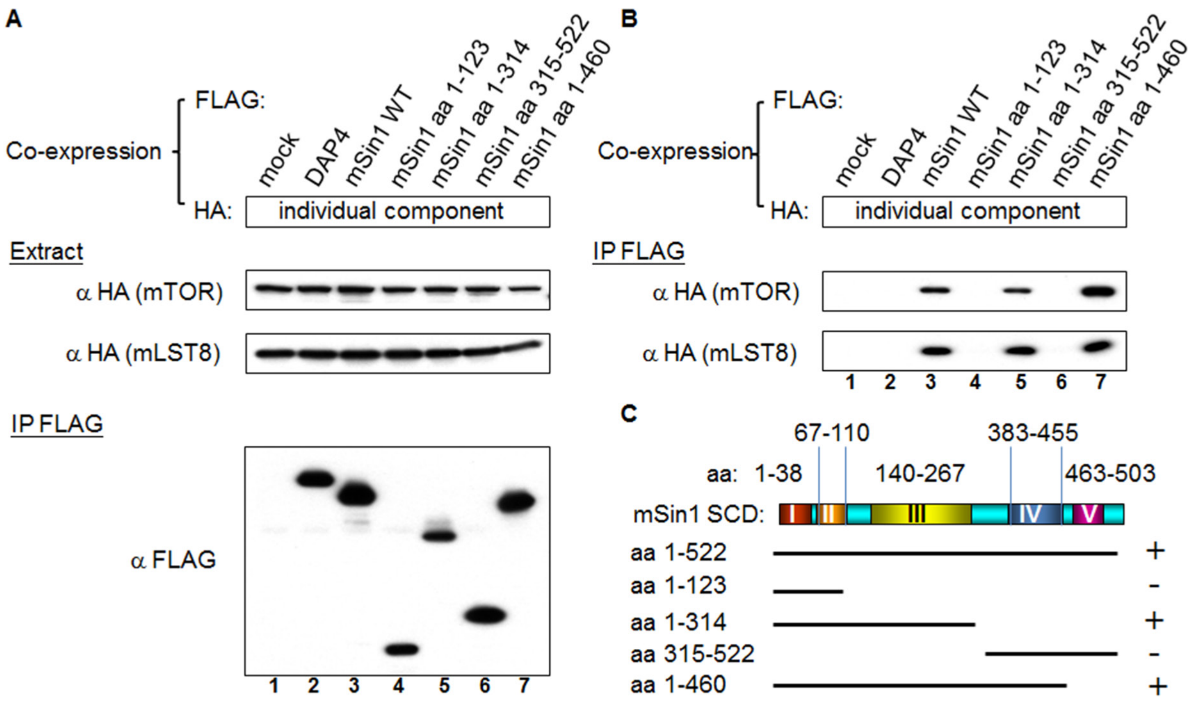

Supplementary Figure 1: mTOR/mLST8 binds mSin1 aa 1-460 and aa 1-314, but not aa 1-123. **(A)** HEK 293T cells were co-transfected with HA-mTOR/HA-mLST8 and the various indicated FLAG-mSin1 fragments. A portion of each extract was used to examine the expression level of the HA and FLAG tagged proteins. **(B)** The majority of each lysate was subjected to FLAG antibody IP. Anti-HA and anti-FLAG antibodies were used to detect the proteins in the total lysate and the associated proteins after the IP. **(C)** The binding between the various fragments of mSin1 and the recombinant HA proteins is summarized here. The recovery of the HA-mTOR and/or HA-mLST8 association is indicated on the right as either + or -. The blots are representative of one experiment repeated twice.

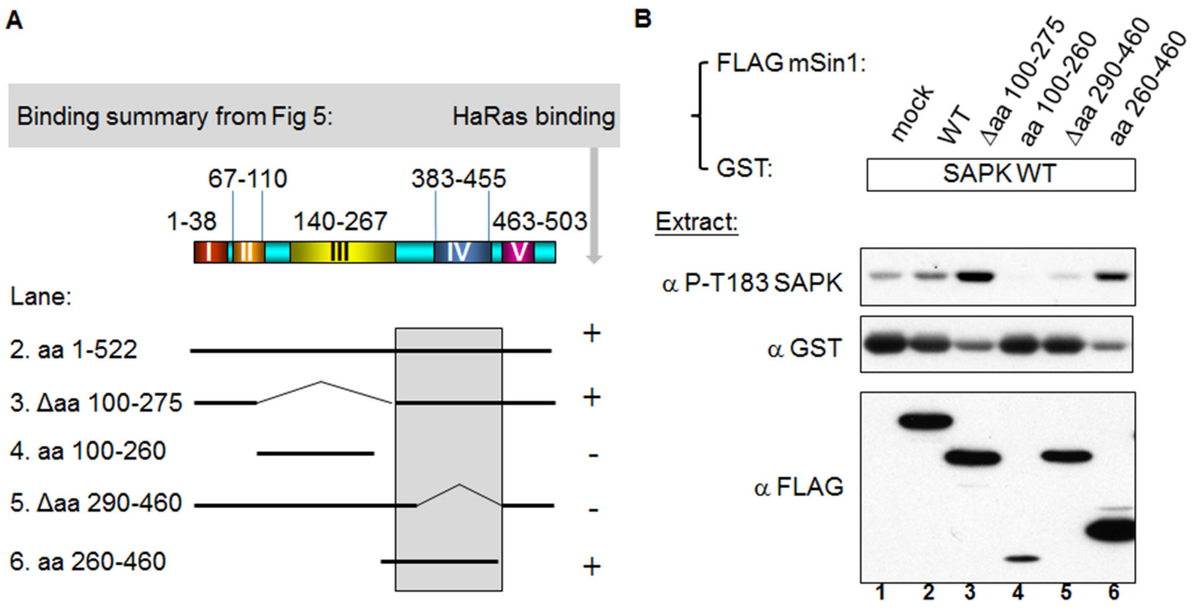

Supplementary Figure 2: The mSin1 aa 260-460, namely the binding sites for HaRas, is necessary for phosphorylation of SAPK at the T183 site. **(A)** The binding between the fragments of mSin1 and HaRas summarized from Figure 5 is simply used as a reference here. The recovery of these proteins, together with the mSin1 fragments, is indicated as either + or -. **(B)** HEK 293T cells were cotransfected with the various indicated FLAG-mSin1 fragments and GST-SAPK WT. Anti-phosphorylated SAPK T183 antibody was used to detect the functioning of these mSin1 fragments.
